# Blowing Kinetics, Pressure Resistance, Thermal Stability, and Relaxation of the Amorphous Phase of the PET Container in the SBM Process with Hot and Cold Mold. Part I: Research Methodology and Results

**DOI:** 10.3390/polym12081749

**Published:** 2020-08-05

**Authors:** Paweł Wawrzyniak, Waldemar Karaszewski

**Affiliations:** 1 Institute of Machine Design Fundamentals, Faculty of Automotive and Construction Machinery Engineering, Warsaw University of Technology, 82-524 Warsaw, Poland; 2 Department of Machine Design and Motor Vehicles, Faculty of Mechanical Engineering, Gdansk University of Technology, 80-233 Gdańsk, Poland; walkaras@pg.edu.pl

**Keywords:** PET, SBM process with hot mold, microcavity, blow kinetics, relaxation of PET amorphous phase, power of ANOVA test

## Abstract

The technology of filling drinks without preservatives (such as fresh juices, iced tea drinks, vitaminized drinks) is carried out using hot filling. Mainly due to the production costs and lower carbon footprint, polyethylene terephthalate bottles, commonly called PET, are increasingly used in this technology. In this paper, the main aim is to describe the statistical analysis methodology of the influence of the temperature of the blow mold in the SBM process and the method of hot filling on the macroscopic and microscopic bottle properties. The macroscopic bottle properties were defined by the thickness profile, pressure resistance, thermal stability, and the coefficients of blowing kinetics. Moreover, the influence of the SBM (stretch blow moulding) process on the microscopic PET material properties (in the bottle) relative to the microscopic preform properties was analyzed. The microscopic properties were defined by the degree of crystallite, density, and relaxation of the amorphous phase of the PET material. For this purpose, response surface experiments were performed for the two analyzed factors (independent variables), i.e., the temperature of the blow mold and the method of hot filling. The sample size was investigated to determine the minimum number of repetitions (number of bottles in the measurement series) required to achieve acceptable measurement uncertainty. The research conducted shows that despite fulfilling the postulate of acceptable measurement uncertainty, in terms of the power of ANOVA (analysis of variance) in DOE (design of experiment) the accepted number of bottles in the measurement series is too small. The tests of the bottle material density, material crystallite, and relaxation of amorphous phase relative to the preform material density, material crystallite, and relaxation of amorphous phase show that the microcavity effects occur during the deformation of the PET material, and that these are associated with the orientation of the microstructure. The blow kinetics study shows that there is a gradient of flow of the bottle material over the thickness of the bottle wall during blowing, and it has been deduced that the air temperature between the blow mold and the wall of the blown bottle has an impact on the kinetics of blowing the bottle.

## 1. Introduction

PET packaging is the most commonly used packaging for storing carbonated and non-carbonated beverages. It involves less energy to produce one PET bottle compared to other packagings, such as aluminum cans or glass bottles. Less energy also translates into a smaller carbon footprint, which nowadays plays a key role from an environmental point of view [1,2].

The technology of filling hot drinks has been used for several years and applies to high-quality drinks without preservatives. In particular, juice, iced tea, or high quality vitaminized drinks should be mentioned here. This is a safe technology because not only the liquid is pasteurized but also the packaging (a bottle and a cap) is sterilized during the filling process. Since the bottle is closed, it is not possible to contaminate the liquid in the bottle with any external contaminants. The filling temperature depends on the type of drink and ranges from 86 to 95 °C.

In the hot filling process, packaging in the form of glass bottles was mainly used in the past. Due to the difficulties in obtaining this type of bottle and to the production costs and environmental protection (smaller carbon footprint), PET bottles now have a growing share in this technology. Nevertheless, the process of pouring into PET bottles is much more complicated due to the properties of the bottles, such as their material structure, the high temperature resistance of PET material, shrinkage prediction, etc. Therefore, research is needed to learn about the behavior of this type of packaging in the process of hot pouring.

A broad review of the literature on the SBM process with cold and hot molds is presented in other articles [3,4,5,6]. For analyzing the impact of changes in the SBM process parameters on the packaging properties, many testing methods have been developed, including tests directly related to the entire SBM process and tests related to individual components of the SBM process (i.e., PET material testing, blowing air flow testing, heat flow testing). Research related to the entire SBM process is [7]:

- free blow of the preform (without blow mold)—enables observations of changes of the shape of the blown bottle during the time of blowing, but only for a limited range of blow pressure (due to cracking of the blown preform) and without capturing the effect of “back pressure” (that is, the air between the blown bottle and the wall of the blow mold) [8,9],
- free blowing of preforms with simultaneous stretching—enables observations of changes of the shape of the blown bottle during the time of blowing with the inclusion of stretching with a stretching rod [8],
- blowing in a transparent blow mold—enables observations of changes of the shape of the blown bottle during the time of blowing, but only for simple bottle shapes, because possible significant curvatures of the transparent mold strengthen the visual distortions [10],
- blowing in the real blow mold (made with aluminum alloy)—enables testing of the impact of SBM process parameters on the quality of finished bottles with complex shapes, but no change in the shape of the blown bottle can be observed during the time of blowing [11,12].

However, in all cases, the material’s movement over the thickness of the blown bottle (blowing kinetics) is defined as being uniform in thickness. In this article, the kinetics of a bottle blown in a real blow mold was also analyzed. The blow kinetics study shows that there is a gradient of flow of the bottle material over the thickness of the bottle wall during blowing, and it has been deduced that the air temperature between the blow mold and the wall of the blown bottle has an impact on the kinetics of blowing the bottle, as is presented in the second part of the article.

It should be emphasized that, in most cases, the statistical analysis of the results of experimental tests does not specify the number of measurement repetitions required so that the measurement results fall within an acceptable error relative to the actual value of the measured feature. Usually three or five repetitions are accepted. This approach seems to be very doubtful, in particular when examining the impact of the SBM process on the properties of manufactured PET bottles. The problem results from the fact that the analyzed sample (PET bottle) is extremely strongly heterogeneous, and the number of repetitions should be determined precisely because of the inclusion of the heterogeneity of the sample in the obtained result. There is also a lack of statistical analysis of the homogeneity of variance and the significance of differences between the research series. It is also not checked in the articles whether the distribution of measurements is a normal distribution, yet analyzing the results on the basis of an arithmetic mean and standard deviation requires that the postulate that the distribution of measurements is a normal distribution is met. In addition, even if a statistical test was used, its power is not checked.

Therefore, the purposes of this article are to:

- define a methodology for determining the number of measurement repetitions so that the measurement error is within acceptable limits relative to the actual value of the measured characteristic,
- define a methodology for calculating the power of ANOVA tests,
- define a methodology for determining the blow kinetics in aluminum blow molds—shear phenomena along the wall thickness of the blown bottle have been noticed, and it has been deduced that the air temperature between the blow mold and the wall of the blown bottle has an impact on the kinetics of blowing the bottle,
- define a methodology for determining the relaxation of the amorphous phase—the occurrence of microcavitation phenomena has been deduced in the PET material of the blown bottle.

Due to the widespread use of polymers for packaging, they are constantly being developed from the point of view of mechanical, thermal, and chemical resistance. Extensive experimental studies on PET bottles in terms of pressure resistance (burst pressure), top load, liquid permeation, and carbonation lost are described in [13]. To optimize the experimental research, the authors used the CCRD method (central composite notable design methods). In statistical analysis, t- and F-statistics were used to test the significance of each model. The research was to determine the optimal amount of RER-PET additive for PET and the concentration of PMDA compound in the RER-PET blend. Research shows that the optimal amount of RER-PET is 20% in terms of pressure resistance and axial load. In the case of liquid permeability and carbonation lost in PET packaging, the optimal amount of RER-PET was determined to be 29%.

The impact of time and the conditions of water storage in PET bottles on the penetration of various chemical compounds into the water was analyzed in [14]. In the statistical study the MDL (method detection limit) values were determined. The MDL value was determined as the average of seven measurements of the concentration of the analyzed substance in clean water (i.e., one in which the concentration of a given factor was certainly zero) and then adding three times the standard deviation of these seven measurements. Another significant value, the instrument detection limit (IDL), is defined as three times the standard deviation of the measurements taken [15]. If the mean values between the measurement series differ less than the value of the three standard deviations for each series, it means that it should not be assumed that these values differ from each other. In experimental studies determining the concentrations of phthalates contaminants in bottled water, the Shapiro-Wilk test was initially carried out to determine the normality of the distribution of results errors, but the number of samples was not determined to guarantee that the measurement result was obtained within the acceptable error [14]. To determine the significance of differences between different times for storing water in PET bottles under the same conditions, the Friedman and Mann-Whitney tests were used, followed by a Mann-Whitney test to determine the significance of differences between different conditions for storing water in bottles.

The impact of wine storage in glass bottles, bottles made of pure (virgin) PET and recycled PET was analyzed in [16]. Each measurement series was repeated three times and one-way ANOVA was used to check the significance of differences in wine storage in different bottles. A Tukey post-test with α = 0.01 was used as a post-hoc test. A similar methodology, but with the value α = 0.05 was used in [17]. It is worth mentioning that some researchers use the Duncan test as a post-hoc test [18].

The amount of antimony in water stored in 12 different types of PET bottles was analyzed in [19]. Three replicate measurements were performed for each type of bottle. The study used a DOE full three-factor, bivalent research plan to determine the most significant factor (storage temperature, water pH, storage time). The research assumed the value α = 0.05, and the results were presented in the form of Pareto charts.

In the tests described in [20], the effect of temperature (four values of the factor) and storage time on the quality of water stored in PET bottles was checked (the quality was determined on the basis of measuring the amount of unfavorable substances in mg that penetrated the water during storage). The measurement series were repeated differently and the number of repetitions was dependent on the series, 2, 3, or 4. The linear regression analysis was performed and ANOVA with α = 0.01 was used to check the significance of differences between the series (due to the very small number of measurement repetitions). If it appeared from the concentration measurements of the test substance that it was lower than the MDL (e.g., for total organic carbon concentration MDL = 0.25 mg/L), these measurements were treated as zero.

The authors of [21] optimized polymer research in the field of the thermo-mechanical degradation of PET. Material degradation was analyzed by MALDI-TOF MS (matrix assisted laser desorption and ionization). Statistical analysis of the experiment (DOE—design of experiment) was used for the three-factor trivalent study. DOE was carried out using the general linear model (GLM), but it should be emphasized that the parameters of the experiments must be carefully considered. To this end, it is necessary to specify the number of measurement repetitions for a given factor value and the factor value change, which must be greater than the signal-to-noise ratio and the resolution of the measuring instrument. In order to check the significance of differences between the series, α = 0.05 was adopted, while the number of repetitions was not defined due to the maximum acceptable error.

The authors of [22] conducted experimental studies on the impact of the geometry of the base of a PET bottle intended for cold filling on the pressure strength, thermal strength (the bottles were heated to 38 °C and stored at this temperature for 24 h), degree of crystallinity, top load strength, wall material distribution of the bottles, environmental stress-crack resistance, and optical properties. Despite a very wide spectrum of measured bottle properties, the results of the measurements were not subjected to any statistical analysis, and moreover no statistical analysis (such as acceptable error, distribution of measurement results, number of repetitions) was determined.

The impact of the SBM process parameters on the quality of manufactured PET bottles in terms of pressure resistance, material distribution on the bottle surface (wall thickness), degree of crystallinity, glass transition temperature, and axial strength was analyzed in [23]. For this purpose, the experiment plan was defined on the basis of a two-factor DOE for the surface temperature of the blow mold (varied from 5 to 50 °C) and the residence time of the bottle in the blow mold (varied from 5 to 20 s). However, the experiment plan was not an overall or symmetrical plan, but it was an optimization plan based on the ECHIP-7 software (ECHIP Inc., Wilmington, DE, USA). The study determined a square optimizing model from which the best values of the blow mold temperature and the residence time of the bottle in the mold were determined, giving the best bottle properties, determined on the basis of the measured characteristics. The research shows that the most favorable residence time of the bottle in the mold is 10.63 s, and the mold temperature is 5 °C. The study did not determine the significance of differences between the measurement series, nor the number of measurement repetitions for specific features determining the quality of manufactured bottles. It should be noted, however, that from the point of view of the technological process both parameters obtained are unacceptable. The residence time of the bottle in the mold is very long, resulting in up to 10 times lower production efficiency, which from the point of view of manufacturing costs is completely unacceptable (standard time is 0.5–1.0 s). A blow mold temperature below 7 °C causes the so-called “retting” of the blow mold (water drops appear on the surface), which is also unacceptable, this time from the point of view of the quality and repeatability of manufactured PET bottles.

The preform temperature, pre-blow pressure, start of the pre-blow relative to the position of the stretching rod, and the amount of air flow during the pre-blow have a significant impact on the quality of the PET bottles produced. The authors of [11] analyzed the impact of these factors on the course of changes in the initial blow pressure inside the blown bottle, the course of force generated on the stretching rod, the kinetics of contact of the wall of the blown bottle with the wall of the blow mold, and on the wall thickness of the bottles produced. A fractional four-factor experiment plan was used there, where the benchmark of the best quality produced was the benchmark for comparing the test results and SBM process parameter values. Nevertheless, the results were not subjected to any statistical analysis and the size of the test sample was not specified.

## 2. Purpose of Research and Methodology of Experimental Research

The literature lacks experimental research on the impact of the SBM process with a hot blow mold on the quality of bottles produced for hot filling. This is due to two problems. The first and most important problem is the strong heterogeneity of the sample, which is the bottle, in all directions and in virtually every analyzed point. This is a problem when determining the location of the sample being cut from the bottle to conduct the standardized DSC (differential scanning calorimetry) analysis, density or mechanical testing. Therefore, one purpose of this article is to define a methodology for determining the number of measurement repetitions so that the measurement error is within acceptable limits relative to the actual value of the measured characteristic. The second problem is the complexity of the SBM process itself with the hot blow mold (a large number of variable parameters).

The main parameters of the SBM process with the hot mold affecting the properties of the hot fill bottle are: The intrinsic viscosity of the preform material, the power profile of the heating lamps in the heating oven (there are seven independent controlled levels of the heating lamps in the heating oven), the heating time in the heating oven (and the associated time of temperature-induced crystallization before the SBM process), the velocity of axial preform stretching, the pre-blow start delay relative to axial preform stretching, pre-blow air pressure, pre-blow time, main blow air pressure, main blow time, heated blow mold temperature profile (there are two heating zones for the blow mold—bottle body zone and bottom zone), the duration of annealing in the hot mold, and the temperature and pressure of the air cooling of the bottle in the blow mold supplied by the stretching rod. Therefore, the properties of a hot fill bottle are influenced by as many as 20 factors in the SBM process with a hot mold.

The intrinsic viscosity of the preform material is very difficult to stabilize because during the injection process the intrinsic viscosity of the preform always decreases relative to the intrinsic viscosity of the raw material. The viscosity of the preform was not included as an independent variable because it cannot be controlled.

In addition, it should be noted that the most independent factors are introduced by the process of heating preforms in a heating oven and heating the bottle surface in a hot blow mold. Therefore, the process of heating preforms in the oven can be minimized from eight factors to one factor, i.e., the overall power of the heating oven. These eight factors relate to seven levels of heating lamps and the overall power setting value of the oven. Conducting tests for all eight factors in an independent manner is very labor-intensive to carry out because the influence of each lamp strongly influences the local kinetics of the bottle during blowing [24]. This is the reason why often in tests one power profile of individual levels of heating lamps is determined, and the impact of the oven is determined by changes in the overall power of the oven [11]. The same can be done with the process of heating bottles in the blow mold and this process can also be minimized from three to two factors, i.e., the overall temperature increase in all heating zones of the blow mold, and the time of heating the bottle in the blow mold. Those three factors relate to the temperature of two heating zones for the blow mold and the duration of annealing in the hot mold.

In total, this constitutes three research factors for the SBM process, and for full bivalent tests, the number of measurement series should be a minimum of eight. However, bivalent tests assume a linear effect of the factor change on the tested properties of the bottle, so a central point should be introduced, with which the linearity postulate can be verified-then nine measurement series will occur.

However, in addition to the SBM process, the properties of the hot fill bottle are also affected by the hot filling process itself. There are three main parameters of the filling process, which affect the properties of the bottle: The time from opening the blow molds to the start of filling, the temperature of the filled liquid, and the annealing time by liquid (the time from the start of filling to the start of the cooling process of the bottle). The impact of changing these parameters will not be analyzed here.

The general goal of future research of the entire SBM process with a hot mold will be to verify the thesis that the relaxation of the amorphous phase has the greatest impact on the thermal stability and pressure resistance of the bottle produced in the SBM process with a hot mold and subjected to the process of hot filling. In addition, it will be checked whether heating the preforms in the heating oven or heating the bottle in a blow mold has the greatest impact on the relaxation of the amorphous phase. In order to analyze the influence of factors (SBM process parameters with hot mold) on the bottle properties, five bottle properties indicators (dependent variables) were adopted. They were:

- bottle thickness profile,
- thermal shrinkage of the bottle (as a macroscopic indicator of the bottle’s thermal stability),
- bottle burst pressure together with the place where the bottle starts to crack (as a macroscopic indicator of the bottle’s pressure resistance),
- blowing kinetics coefficients (as indicators of preform material displacement during the SBM process),
- degree of relaxation of the amorphous phase (as a microscopic indicator of relaxation of the oriented amorphous phase).

Due to the fact that the SBM process with a hot mold is an extremely complex process and the bottle has many features, it is not possible to include all the tests, and thus the description of the methodology, in one article. This article describes the methodology for measuring the adopted dependent variables, and preliminary statistical tests of the SBM process with a hot mold, verifying assumptions about the normal distribution of the measurement error and determining the sample size for individual dependent variables. The second part of the article compares the features of the cold fill bottles (manufactured in a cold mold) with hot fill bottles (manufactured in a hot mold).

**Figure 1:**
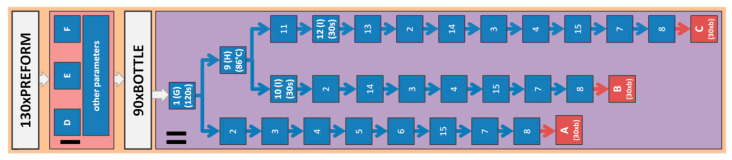
The steps for producing bottles for testing the analysis of assumptions about the normal distribution of measurement errors, and determining the sample size; designations are explained in Table 1.

All the tests involving bottles were done using a linear blow molding machine Blueline 8 HiTech [25]. All the analyzed bottles were made on the same cavity of the mold. Figure 1 shows schematically the individual steps for producing bottles for testing the analysis of assumptions about the normal distribution of measurement errors and determining the sample size. Based on the knowledge of the authors of this study, it was assumed that 130 bottles were produced in tests verifying the normal distribution of errors, with the first 35 and the last 5 rejected. The rejection of the initial bottles was intended to stabilize the SBM process, and the rejection of the last ones was to eliminate the negative impact of the heating oven on the extreme bottles in the series. The remaining 90 bottles were divided, depending on the filling process, into three measuring series A, B, and C of 30 bottles each (variance for the population can be estimated using an *n*-element random sample, and for a sample size of *n* = 30, the uncorrected estimator gives the same results as the corrected estimator), as shown in Figure 1. The designations used in Figure 1 are explained in Table 1

Research on error and determination of the sample size for selected parameters of selected bottle features carried out for the parameters of the SBM process and the hot filling process:

- D—General power of heating lamps in the preform heating furnace: 65%,
- E—General power of heating heaters in a hot blow mold for hot mold: 60%, or water cooling temperature blow mold for cold mold: 10 °C,
- F—Time of annealing the bottle in a hot blow mold, or the time of bottle staying in a cold blow mold: 1.5 s,
- G—Time from opening a blow mold to starting filling process: 120 s,
- H—Water filling temperature: 86 °C,
- I—Annealing time with hot water: 30 s,
- Hot water heating method: Lack (A series), free (B series), bath (C series).

**Figure 2:**
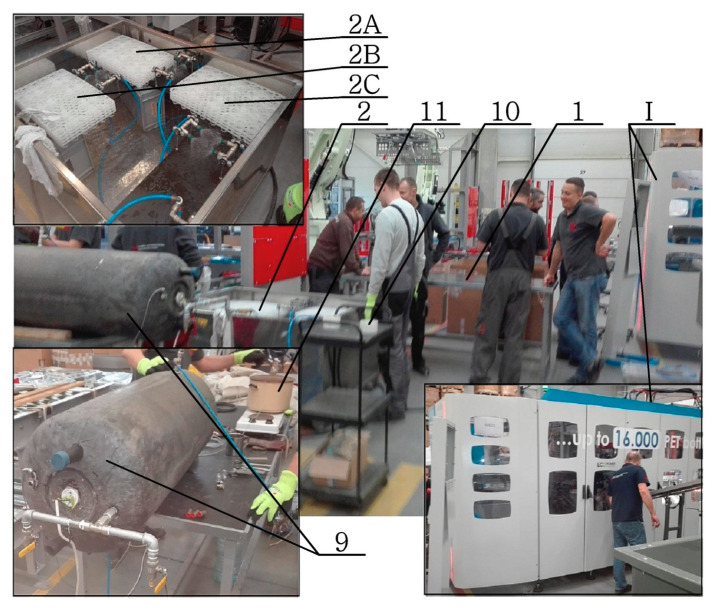
Measuring station for filling, annealing, and cooling of manufactured bottles (the numbering shown in the figure refers to the numbering shown in Figure 1 and Table 1).

A photo of the measuring station for filling, annealing, and cooling of the bottles produced in the SBM process is shown in Figure 2, where the numbering shown refers to the numbering shown in Table 1.

Table 2 shows the values of other SBM process parameters not treated as independent variables in the research. The parameters of the SBM process that were not treated as independent variables, i.e., for all tests, had the same values, namely: Stretching rod speed (axial preform stretching), pre-blow start delay relative to the position of the stretching rod, pre-blow air pressure, pre-blow time, main blow air pressure, main blow time, cooling air temperature in the blow mold, blow mold temperature profile, post-mold bottles cooling air pressure and temperature, and heating profile power of individual heating lamps as a percentage of their maximum power.

**Figure 3:**
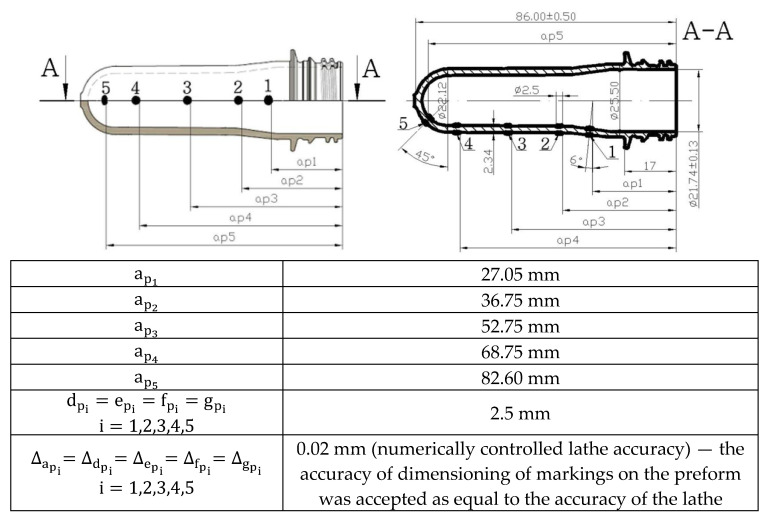
The dimensions of the preform used in the tests, as well as the location and dimensions marking applied to the outer and inner walls of the preform, used to define the coefficients of the bottle blow kinetics.

**Figure 4:**
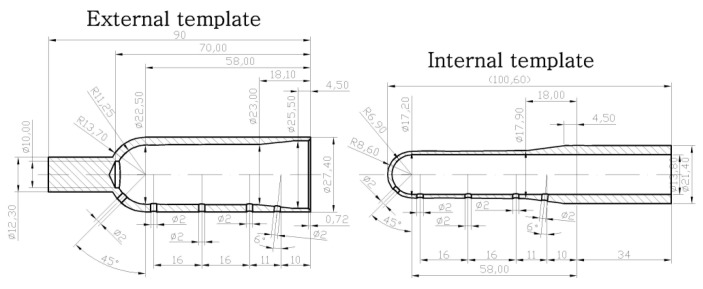
Dimensions of templates used for marking, marked on the outside and inside walls of the preform, used to define the coefficients of the bottle blow kinetics.

**Figure 5:**
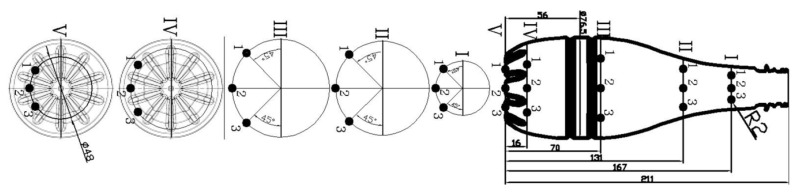
The shape of the analyzed bottle and the location of individual measuring points of the bottle wall thickness, also showing the area of thickness measurements around the measuring points for the example of point I-3 (explanation in Figure A1). The determination of the axial position of the point will be explained in the second part of this article.

The drawing of the tested preform is shown in Figure 3. Figure 3 also shows the location of the five external and internal markings, and their dimensions, which are the basis for the preform definition of the coefficients of the bottle blowing kinetics. The external and internal markings were made using the templates shown in Figure 4. Figure 5 shows the shape of the bottle being analyzed and cross-sections, where I, II, II, IV, and V marking points were placed. In each cross-section three measuring points are located circumferentially, in which the thickness was measured. The most likely value of absolute thickness measurement uncertainty is shown by Formula (A1).

In Formula (A1), the problem is to determine the extension factor “k” because it has a value resulting from the accepted level of confidence and the distribution resulting from the composition of normal distribution (type A uncertainty) and uniform distribution (type B uncertainty). The resultant distribution is a convolution of the constituent distributions and its determination creates many problems. For this reason, a method enabling an approximate determination of the expansion coefficient should be used, which is based on the assumption that the resultant distribution coincides with a distribution with a larger standard deviation [26]. If (uncertainty of type A) > (uncertainty of type B), then the expansion coefficient assumes the values of a standardized random variable of the normal distribution (or t-Student). If (uncertainty of type A) < (uncertainty of type B), then the coefficient k assumes the values characteristic of a uniform distribution. It was assumed in the study that for all cases the value of the coefficient “k” is equal to 2.

**Figure 6:**
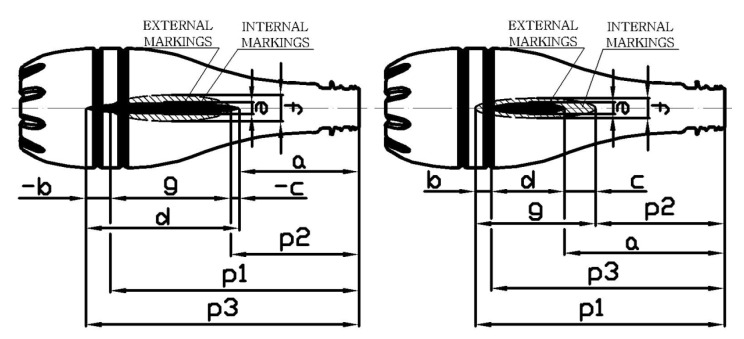
A way of measuring the marks that are the basis of the bottle kinetics analysis.

Figure 6 shows how to measure the dimensions that are the basis of the bottle to define the coefficients of the bottle blowing kinetics. The bottle blowing kinetics coefficients have been defined as the quotient of the kinetics coefficients of the bottle (Figure 6) by the corresponding kinetics coefficients of the preform (Figure 3 and Figure 4). The coefficients of the bottle blow kinetics were calculated on the basis of the dimensions shown in Figure 6, and their formulas are shown in Equations (A2), (A4), (A6), (A8), (A10), (A12), and (A14). Equations (A2)–(A21) lists the coefficients of the bottle blowing kinetics, and the method calculating them on the basis of the measurement variables (a, p1, p2, p3, e, f), along with showing how to calculate the most likely measurement uncertainty value for the coefficients of the bottle blow kinetics (A3), (A5), (A7), (A9), (A11), (A13), and (A15), respectively.

The relative thermal shrinkage of the bottle was calculated as the quotient of the maximum volume difference before and after the water annealing process of the produced bottle by the maximum volume before the water annealing process. However, it should be mentioned that the value of the bottle’s thermal shrinkage (which is a measure of the bottle’s thermal stability) can be affected by the weight of the water stored inside the bottle, i.e., the hydrostatic pressure exerted by the water can counteract the reduction in the bottle volume, which will distort the PET’s response to the elevated water temperature. Therefore, it should also be checked whether this effect is significant, and the bottle shrinkage test by water annealing was carried out in two ways. The first method, called free annealing (Formula (A22)), reflects the actual technological process of the hot filling process, and involves filling hot water into a bottle and filling it out for a period of time. In contrast, the second method of water annealing, called bath annealing (Formula (A24)), reduces the impact of the water mass pressure on the thermal response of the bottle, by placing the bottle filled with hot water into the hot water tank and keeping it in that bath for a period of time. However, the second method of testing shrinkage, despite the fact that it more accurately determines the response of the bottle material itself to heat from hot water, differs from the actual technological process occurring in the industry.

Due to the fact that there were two ways of water annealing, the bottle shrinkage was also calculated for each annealing method separately, and so for free annealing, the relative bottle shrinkage was calculated according to Formula (A22), and for bath annealing, according to Formula (A24). The most probable measurement uncertainty value was defined by Formulas (A23) and (A25) for free annealing and bath annealing, respectively.

The microscopic amount of “oriented” and “rigid” amorphous phases (as the inverse of the measure of relaxation of the amorphous phase) in a multiphase PET model can be estimated indirectly using a two-phase PET model (the amorphous-crystalline model of PET). Knowing the density of crystallites and the density of the “non-oriented” amorphous phase of a PET material, the amount of “oriented” and “rigid” amorphous phase can be determined on the basis of a precise measurement of the density of the material. The idea of indirect measurement is that the DSC (differential scanning calorimetry) method determines the weight degree of crystallization of the material (according to Formula (A32) [27]). Then, by examining the density of the PET sample and using Formula (A33) [27] for the weight degree of crystallinity of the material determined by the density measurement method (see Table 3), and assuming that it is equal to that calculated from the DSC method, the average density of the amorphous phase (oriented, rigid, and non-oriented) can be determined according to Formula (A34). The degree of relaxation of the amorphous phase was defined by Formula (A36) as the quotient of the difference of the mass of the amorphous phase with the measured density of the amorphous phase and the mass of the amorphous phase with the density of the non-oriented amorphous phase by the mass of the amorphous phase with the measured density of the amorphous phase, assuming that the volumes of each of these masses are equal. The most likely value of measurement uncertainty was shown by Formulas (A35) and (A37) for the density of the amorphous phase and the degree of relaxation of the amorphous phase, respectively.

The adopted method of microscopic determination of the degree of relaxation of the amorphous phase captures the fact that the amorphous phase is ordered only “to a certain maximum degree” that characterizes the crystalline phase. In other words, all phases that are not ordered as a crystalline unit are treated as the average measure as non-crystalline phases. There is no fixed parameter such as “oriented phase density” or “rigid phase density” as the set value for PET. The density of these phases depends on the degree of orientation and conformation of the chains and varies from the density of the amorphous unoriented phase (zero orientation) to full orientation (the density of the crystalline phase). The determination of the average density of the amorphous phase (according to Formula (A34)) is based on the assumption that there is no amorphous non-oriented phase. The entire non-crystalline phase is treated in some way as the ordered amorphous phase, and not as a proportion of ordered fraction with a fixed density. Then, this average density of the amorphous phase will be a measure of the amount of the non-crystalline phase, and thus a measure of the degree of relaxation of the amorphous phase (defined by Formula (A36)).

However, it should be also noted that samples used to measure the density and weight crystallization degree were cut out of bottles at the IV-2 thickness measurement point (base part of the bottle shown in Figure 5), and cut out of preforms at four blow kinetics marks (shown in Figure 3). There are huge differences in the crystallinity of various parts during the molding of polymer materials, but in the article it was focused on the orientation of amorphous phase so it was decided to measure the microstructure only in the area which is the most deformed after blowing and during the hot filling process (i.e., the base part of the bottle). What is more, the base part of the hot blow mold is heated only to 62 °C (see Table 2) so the crystallization process is substantially slowed after blowing in comparison with the label part of the hot blow mold (125 °C)—so the orientation of the amorphous phase should be more visible. In theory, the statistical analysis should have to be performed on both of these bottle’s parts, nevertheless the microstructure of the label part of the bottle should not have been done due to the fact that this sample would be too crystallized. 

In addition, the bottle was tested for pressure resistance. The test consisted of pumping water inside the bottle, and uniformly increasing the pressure of that water until the bottle burst. The water pressure at which the rupture occurred was a measure of the pressure resistance of the bottle. The most likely value of the measurement uncertainty in the measurement of the pressure resistance is shown by Formula (A40).

It should be emphasized that of all the bottle features measured, some of the measurements were destructive and some were non-destructive measurements relative to one bottle. Destructive measurements in terms of the bottle include pressure resistance measurements (only one measurement can be repeated for one bottle), whereas non-destructive measurements in terms of one bottle include thickness measurement, measurement of dimensions needed to calculate the blow kinetics coefficients, and maximum volume measurement (multiple repetitions can be done for one bottle). Measurement of the density in the gradient column and the degree of crystallinity by the DSC method is carried out on samples cut out from the bottle (so they are destructive measurements relative to one bottle). For the excised sample, it is possible to perform many repetitions of density measurement in a gradient column (it is a non-destructive measurement due to the excised sample) and only one degree of crystallinity measurement by the DSC method (it is a destructive measurement due to the excised sample). Although many samples lying close to each other can be cut from one bottle, therefore it is theoretically possible to perform many repetitions of density and crystallinity measurements for one bottle, the research revealed that the microstructure of the bottle is so heterogeneous that DSC measurements of crystallinity cannot be used for different samples (cut even close together) as a repeat of the measurement for one bottle.

Due to the fact that all measurements of output quantities will be carried out after the SBM process under isothermal conditions, the speed of operation of measuring instruments is not important in the context of measurement accuracy—the test is static. A summary of information on the methods and measurement tools used for individual dependent variables is presented in Table 3.

There is a problem with the so-called outliers, i.e., measurements that are very different from the other points in a given measurement group. Such measurements may disrupt parametric inference even with corrections for the heterogeneity of variance. Prior to the statistical analysis, the measurement database was verified for the occurrence of extremes and outliers (thick errors) using the Grubbs test [28] or box-and-whisker plots for measuring the density and degree of crystallinity by the DSC method. 

**Table 3:** A summary of information on methods and measurement tools used for individual dependent variables—for the maximum measurement error a rectangular error distribution was assumed for the entire measurement range.

Features	Dependent Variables					
	Thickness Profile	Blow Kinetics Coefficients	Bottle Weight	Pressure Resistance	Degree of Crystallinity ^1^	Density ^1^
Method	Measurement of bottle thickness at selected point (Figure 5)	Measurement of the dimensions of the measuring points shown in Figure 3, Figure 4, Figure 6	Measuring the weight of an empty bottle and filled with maximum fill level water	Bottles burst test with water pressure	DSC analysis at a rate of 10 °C/min	Measurement in accordance with ASTM D 1505-85 norm
Measurement tool	Inductive sensor FH4 [29]	Electronic altimeter, an electronic caliper	Electronic scale	CMC KUHNKE ABT-3100-PET [30]	TA Inst Q20 microcalorimeter	Gradient column ^2^
Meter type	digital meter	digital meter	digital meter	digital meter	digital meter	analog meter
Sample	bottle	bottle	bottle	bottle	1 × 1 cm square sample cut out from base area IV-2 point (Figure 5)	1 × 1 cm square sample cut out from base area IV-2 point (Figure 5)
Maximum measurement error	$\Delta_{1}=\pm 0.005\;\mathrm{mm}$	altimeter $\Delta_{2}=\pm 0.01\;\mathrm{mm}$ caliper $\Delta_{7}=\pm 0.01\;\mathrm{mm}$	$\Delta_{3}=\pm 0.5\;g$	$\Delta_{4}=\pm 0.1\;\mathrm{bar}$	$\Delta_{5}=\pm 2\%$	$\Delta_{6}=\pm 8.3\cdot 10^{-5}\;g/{\mathrm{cm}}^{2}$

^1^ All microstructure measurements were made in the Polymers Division of The Centre of Molecular and Macromolecular Studies in Łódź. ^2^ Gradient column filled with water solutions of calcium nitrate (in the range from 1.32 to 1.42 g/cm^3^)—measurements were carried out at temperature 25 °C.

Before testing the impact of the SBM process parameters and the hot filling process on the behavior of bottles in the hot filling process, statistical tests were carried out to verify the assumptions about the normal distribution of the measurement error and to determine the sample size for individual tests, i.e., for the test of thermal stability (maximum bottle volume test), thickness profile, pressure resistance, blow kinetics coefficients, and relaxation of the amorphous phase. Then, the sample size was calculated (according to Formula (1) [31]—the formula for the sample size for the mean) for measuring the thickness profile, dimensions needed to calculate the blow kinetics coefficients, pressure resistance, empty and water-filled bottle, degree of crystallinity, and density of the bottle material.

(1) $$n={\left(\frac{Z_{\alpha /2}\cdot \sigma_{p}}{\Delta_{\max}}\right)}^{2}=\left\{for\;\alpha =0.05\to Z_{\alpha /2}=1.96\right\}={\left(\frac{1.96\cdot \sigma_{p}}{\Delta_{\max}}\right)}^{2}$$

where $\Delta_{\max}$ is the desired level of precision (in the same unit of measure as the variance)—acceptable measurement uncertainty (maximum acceptable error); n is the number of repetitions of measurements, the sample size; $\sigma_{p}$ is the variance of an attribute in the population; $Z_{\alpha /2}$ is the abscissa of the normal curve that cuts off an area α at the tails (for normal distribution $Z_{\alpha /2}=1.96$).

## 3. Preliminary Statistical Research

**Figure 7:**
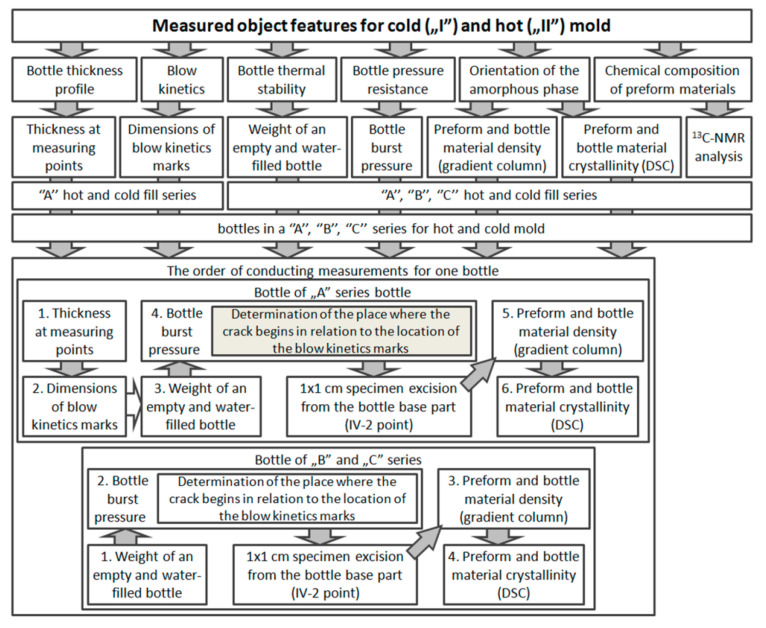
The order of testing the thickness profile, dimensions of the markers of the kinetics of blowing, thermal stability, pressure resistance, and relaxation of the amorphous phase of the bottle intended for cold and hot filling.

**Figure A1:**
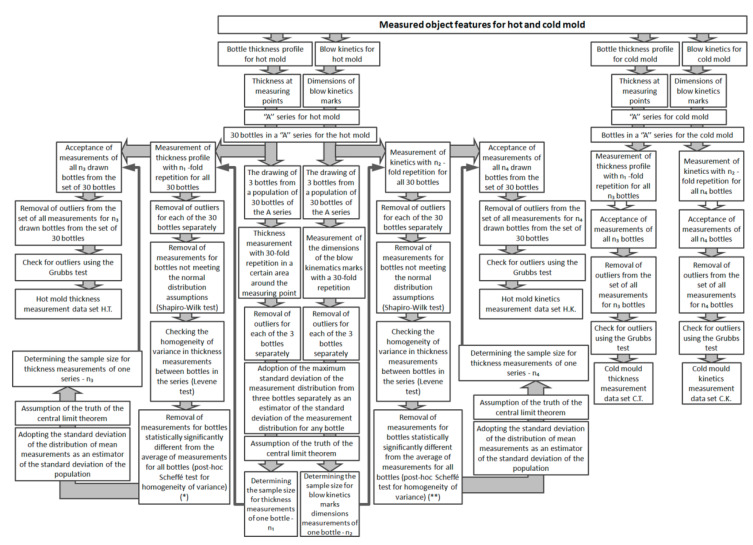
A method of statistical preparation of a set of measurement data for bottles for cold and hot filling, respectively for thickness (C.T., H.T.) and dimensions of blow kinetics determinations (C.K., H.K.) (sample size was determined in accordance with Formula (1)); (*) data used to determine the process stabilization in terms of thickness profile, (**) data used to determine the process stabilization in terms of blow kinetics determinations. The specific area around the measuring point (with radius 2 mm) in which the thickness measurement with a 50-fold repetition was done as shown in Figure 5 for the I-3 point as an example.

**Figure A2:**
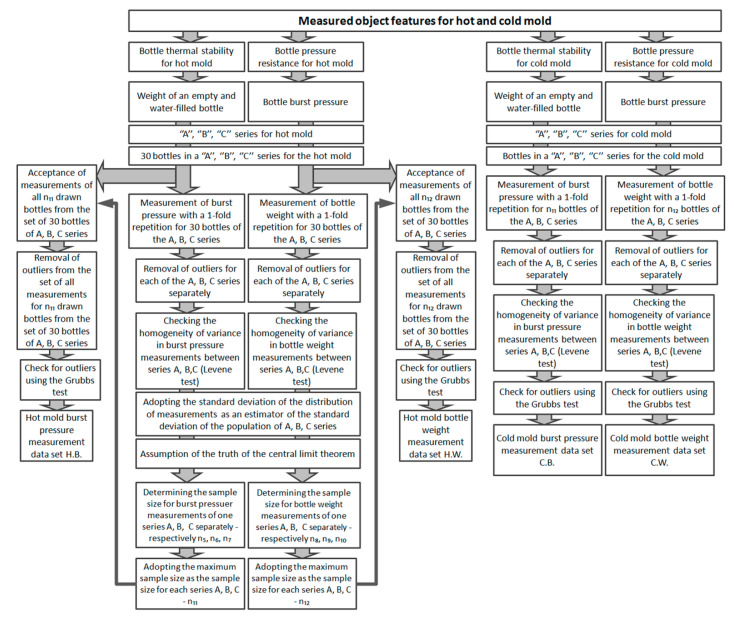
Method of statistical preparation of the measurement data set for pressure resistance (H.B.) and thermal stability (H.W.) of hot fill bottles as well as pressure resistance (C.B.) and thermal stability (C.W.) of cold fill bottles, respectively (the sample size was determined in accordance with Formula (1)).

**Figure A3:**
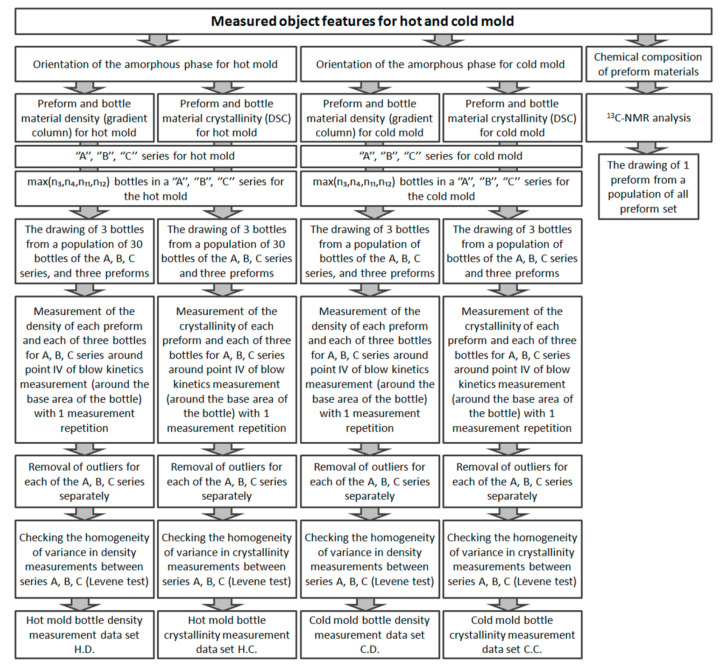
The method of statistical preparation of a set of measurement data for the degree of crystallinity by the DSC method (H.C.) and density in the gradient column (H.D.) of hot fill bottles and the degree of crystallinity by the DSC method (C.C.) and density in the gradient column (C.D.) of cold fill bottles, respectively.

Figure 7 shows the order of testing the thickness profile, the dimensions of the markers of the blowing kinetics, and the thermal stability, pressure resistance, and relaxation of the amorphous phase of the bottle intended for cold and hot filling. Figure A1, Figure A2 and Figure A3 show the method of statistical preparation of a set of measurement data for the thickness profile and dimensions of the markers of the blowing kinetics of hot and cold fill bottles (Figure A1), the thermal stability and pressure resistance of a hot and cold fill bottle (Figure A2), and relaxation of the amorphous phase of the hot and cold fill bottles (Figure A3). The removal of outliers consisted of rejecting measurements that go beyond 1.96 standard deviation of the sample from the average value of the given sample. It is worth mentioning that if a lot of bottles differ statistically from the average value (according to the post-hoc analysis of the Scheffé test when the homogeneity of variance is fulfilled or the Duncan test when the homogeneity of variance is not fulfilled) for measuring the thickness profile and the coefficients of the blow kinetics, it will mean that the process has not been fully stabilized and there are strong differences between the bottles in the analyzed A series.

## 4. ANOVA Test Power Calculation

In the preliminary statistical research, two types of tests were performed to verify the measurement population for the occurrence of outliers. The first was the Box Plot chart, i.e., if a given measurement exceeds the range of 1.96 times the standard deviation from the mean value, it is treated as an outlier measurement and removed from the measurement population. The second test was the Grubbs test. In order to check the normal distribution of the measurement results, the Shapiro–Wilk test was carried out. Levene’s test was performed to check the homogeneity of variance. In order to check the statistically significant difference between the analyzed measurement series, ANOVA tests were carried out. Depending on whether the given measurement group met the requirements for parametric tests or not, the post-hoc tests were performed, respectively the Scheffé test for parametric tests and the Dunnett test for non-parametric tests. Then, the DOE (design of experiment) analysis was performed for the analyzed two factors (independent variables), i.e., the temperature of the blow mold, and the hot filling method. The mold temperature had two values, while the filling method had three values. The tested bottle features (dependent variables) were the thickness profile, blowing kinetics, pressure resistance, thermal stability for bottles; also, the density, degree of crystallinity, and relaxation of the amorphous phase, but between the preform and bottles. All tests were carried out in the environment of Statistica 13.0.0.0 Copyright 1984–2017, TIBCO Software Inc., Palo Alto, CA, USA.

In the DOE analysis, an experiment plan must first be built in order to be able to determine the effect values in the adopted model. In this work, a linear model (with constant coefficients) was adopted, where the effect value corresponds to the coefficient value for a given independent variable. Assuming a two-factor plan, Formula (2) shows the linear model for which the values of the coefficients $b_{1}$, $b_{2}$, $b_{3}$ must be calculated, determining the relationship between the values of independent variables $A_{i}$, $B_{j}$, and the answer $y_{\mathrm{ijl}}$ (dependent variable). The values of the coefficients $b_{1}$, $b_{2}$, $b_{3}$ are determined on the basis of minimizing the total model error (Formula (3)), e.g., by the method of least squares [32]. Additionally, ANOVA models assume that the responses $y_{\mathrm{ijl}}$ within each cell are an independent and identically distributed random normal with a constant variance *σ*e^2^ (model (3)) [33].

However, non-standardized regression coefficients cannot be compared with each other directly due to the different measurement units and different variances of the explanatory variables. Table 4 and Equations (2)–(4) shows the plan of a two-factor, bivalent experiment. Therefore, it is necessary to standardize the variables to obtain a constructive comparison. Knowing the coefficients of Equation (2), then this equation can be presented in a standardized form using a standardized effect estimate. Equation (2) using a standardized effect estimate is shown by formula (4), where $\sigma_{y}$, $\sigma_{A}$, $\sigma_{B}$, $\sigma_{\mathrm{AB}}$ are the standard deviations of the test results (dependent variables) and independent variables A, B, AB, respectively, while $\bar{A}$, $\bar{B}$, $\bar{\mathrm{AB}}$ are the arithmetic means of the values of independent variables A, B, and AB, respectively. In the simple linear regression, the value of the standardized regression coefficient is exactly the same as the correlation coefficient; its meaning can be interpreted in the same way [34].

(2) $$y_{\mathrm{ijl}}=\tilde{y}+b_{1}\cdot A_{i}+b_{2}\cdot B_{j}+b_{3}\cdot \left(A_{i}\cdot B_{j}\right)+\varepsilon_{\mathrm{ijl}}=\mu_{\mathrm{ijl}}+\varepsilon_{\mathrm{ijl}}$$

(3) $$\varepsilon =\overset{N_{\mathrm{ij}}}{\underset{l=1}{\sum }}\overset{2}{\underset{i=1}{\sum }}\overset{w}{\underset{j=1}{\sum }}\varepsilon_{\mathrm{ijl}}{}^{2}=\overset{N_{\mathrm{ij}}}{\underset{l=1}{\sum }}\overset{2}{\underset{i=1}{\sum }}\overset{w}{\underset{j=1}{\sum }}{\left(y_{\mathrm{ijl}}-\mu_{\mathrm{ijl}}\right)}^{2}\;\mathrm{with}\;a\;\mathrm{distribution}\;N\left(0,\sigma_{e}{}^{2}\right)$$

(4) $$\frac{y_{\mathrm{ijl}}-\tilde{y}}{\sigma_{y}}=\frac{b_{1}\cdot \sigma_{A}}{\sigma_{y}}\cdot \left(\frac{A_{i}-\bar{A}}{\sigma_{A}}\right)+\frac{b_{2}\cdot \sigma_{B}}{\sigma_{y}}\cdot \left(\frac{B_{j}-\bar{B}}{\sigma_{B}}\right)+\frac{b_{3}\cdot \sigma_{\mathrm{AB}}}{\sigma_{y}}\cdot \left(\frac{A_{i}\cdot B_{j}-\bar{A\cdot B}}{\sigma_{\mathrm{AB}}}\right)$$

However, even if a model is built, it is still necessary to check whether it significantly reflects the impact of the independent variables on the dependent variables. To this end, ANOVA has to be performed. It is worth remembering that the ANOVA analysis of variance can be performed for both independent and dependent tests (repeated measurements). In addition, it is a parametric test, so it is worth bearing in mind the assumptions of such tests: The normality of the distribution of the dependent variable in the compared groups, equal variances, equal groups, and at least the interval measurement level of the dependent variable. A description of multifactorial ANOVA can be found, e.g., in [35].

In the ANOVA analysis a certain level of acceptable error of the first type (α) should be assumed, but it does not specify whether the analysis has adequate statistical quality. In general, the quality of ANOVA statistical analysis in DOE depends on the following factors:

- the adopted level of the first type of error (the higher this is, the higher is the quality of the analysis, but also the likelihood of making the first type of error increases, as a result of which the certainty of rejecting the null hypothesis decreases);
- the variance of the distribution of measurements in the test sample, which is influenced by the number of measurement repetitions, error from the measuring instrument, error from the researcher (the higher the variance of the distribution, the lower the quality of the analysis);
- maximum difference between main effect means;
- number of factor levels in the model.

In each statistical test, a hypothesis is formulated at the first stage, which is subject to verification and this tested hypothesis is called the null hypothesis—H0. The null hypothesis is formulated in such a way that it can be rejected on the basis of the test results. In addition, an alternative hypothesis is formulated—H1. It is assumed that the null H0 hypothesis is true.

Two types of errors can be made when verifying hypotheses. The error of rejecting the tested true null hypothesis is called the first type error (error α). The error consisting of adopting the tested false null hypothesis is a second type error (error β). The level of significance, marked with the symbol α, is chosen in advance as the probability of making the first kind of error. Rejection of the tested null hypothesis at the significance level of α = 0.05 means that the risk of making the first kind of error with this decision was 5%. The power of the test is the probability of failure of the second type error (understood as the difference in certainty and the likelihood of making a second error of type β—Formula (5)), consisting of adopting the false null hypothesis (not rejecting the null hypothesis, which is actually false).

(5) Power=1−β

The recommended level of significance is 0.05, and the statistical testing power level is 0.8. The smaller the second type of error, the more powerful the test. Increasing the level of significance, increasing the sample size, and improving the accuracy of measurements (e.g., by increasing the sensitivity of the measuring instrument) all improve the test power. However, increasing the level of significance also increases the probability of making the first type of error.

**Figure 8:**
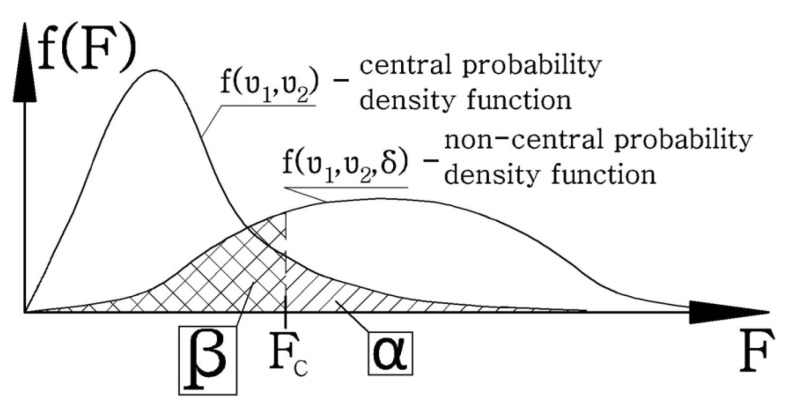
Method of determining the probability of making a second type error (β) in DOE analysis based on the example of the analysis of the effect of the independent variable on a dependent variable.

In most articles, the test of the power of statistical tests used is omitted due to the difficulty in calculating the second type of error (β). Figure 8 shows the method of calculating the II-type error in the ANOVA analysis; for this purpose the probability for the non-central F distribution (with a non-zero non-central parameter *δ*) should be calculated, for the critical value Fc corresponding to the probability of making the error of the first type (α) for the central F distribution.

While the central F distribution characterizes how the F test statistic is distributed when the null hypothesis is assumed to be true, the non-central F distribution instead shows how the F test statistic is distributed when the alternative hypothesis is assumed to be true (i.e., when the null hypothesis is assumed to be false). As such, it is useful in calculating the power of the usual F tests (ANOVA, regression, etc.) [36]. 

Knowing the value of the probability of making a second type error (β), it is possible to calculate the test power according to Formula (5). It was assumed that the minimum power cannot be lower than 80%, i.e., the probability of making a second type error cannot exceed 20%.

In the case of analysis of the non-central distribution F, the big problem is the calculation of the non-central parameter *δ* (which is calculated from the Root Mean Square Standardized Effect (RMSSE)—Formula (6)). The RMSSE is the square root of the sum of squared standardized effects divided by the number of degrees of freedom for the effect. For individual main effects [33] and two-factor ANOVA interactions, this leads to Formulas (7)–(9), where ${\mathrm{SS}}_{A.\mathrm{ef}}$, ${\mathrm{SS}}_{B.\mathrm{ef}}$, ${\mathrm{SS}}_{\mathrm{AB}.\mathrm{ef}}$ are respectively the sum of the squares of the mold main effect, hot fill main effect, and interaction effect. The variance *σ*e^2^ is the highest possible probability of the measurement uncertainty value of the measurement for the test object in the measurement series (calculated according to Formula (10)).

(6) $$\delta_{\mathrm{ef}}=N_{\mathrm{ef}}\cdot {\mathrm{df}}_{\mathrm{ef}}\cdot \mathrm{RMSE}E_{\mathrm{ef}}{}^{2}.$$

(7) $$\delta_{A.\mathrm{ef}}=\frac{{\mathrm{SS}}_{A.\mathrm{ef}}}{\sigma_{e}{}^{2}}$$

(8) $$\delta_{B.\mathrm{ef}}=\frac{{\mathrm{SS}}_{B.\mathrm{ef}}}{\sigma_{e}{}^{2}}$$

(9) $$\delta_{\mathrm{AB}.\mathrm{ef}}=\frac{{\mathrm{SS}}_{\mathrm{AB}.\mathrm{ef}}}{\sigma_{e}{}^{2}}$$

(10) $$\sigma_{e}{}^{2}=\max\left({\left(\sigma_{e}{}^{2}\right)}_{\mathrm{ij}}\right)\;\mathrm{where}\;i=1,2;\;j=1,2$$

A summary of the measurement errors Δ of the measurement tools used is shown in Table 3. For direct measurements, with uniform distribution of the density function of the probability density of the error distribution of the measuring instrument Δ, and an arbitrarily accepted confidence level *p* = 0.95, the variance from Formula (10) was calculated from Formula (A41) (for the thickness profile) and from Formula (A42) (for pressure resistance, degree of crystallinity in the DSC method, and density in the gradient column). The problem is to determine the measurement variance (10) for testing the shrinkage (thermal stability), blow kinetics coefficients, and relaxation of the amorphous phase (defined by Formula (A36)), because the measurement is not made directly but indirectly using either electronic scales, calipers, an altimeter or DSC apparatus, and a gradient column. The variation of the measurement error for the shrinkage test can be calculated from Formula (A50), for the test of blow kinetics coefficients from Formulas (A43)–(A49), and for the relaxation of the amorphous phase from Formula (A51).

## 5. Plan of Experiments and Result

Table 5 shows the plan for one one-factor bivalent (1 × 2) experiment for the SBM process, where the first value of the factor was the cold mold temperature and the second value of the factor was the hot mold temperature for the “A” series of the hot filling method. For this plan, studies on the thickness profile and blow kinetics were carried out. 

Table 6 shows the plans for six one-factor bivalent (1 × 2) experiments for the SBM process with the hot fill process, where the first value of the factor was the preform feature and the second value of the factor was the bottle feature produced in the SBM process with a combination of two mold temperatures (cold, hot) and three hot filling methods (A, B, C). Studies on the degree of crystallinity, density, and relaxation of the amorphous phase were performed for each of the six plans. 

Table 7 shows the plans for nine one-factor bivalent (1 × 2) experiments for the SBM process or hot fill process. Studies on the pressure resistance and thermal stability were performed. Moreover, three two-factor bivalent plans were adopted, in which a linear model with two main effects and one two-factor interaction effect was implemented separately. The plans of three two-factor bivalent (2 × 2) experiments for mold temperature and three combinations of hot filling, i.e., without hot filling (A) and free annealing (B), without hot filling (A) and bath annealing (C), with free annealing (B) and bath annealing (C), are presented in Table 8. For each plan, bottle pressure resistance tests were carried out, and, for one, additional thermal stability tests. 

For every plan, the power of ANOVA statistical test was calculated. Interpretation and statistical analysis of the results is described in the second part of this work.

## Figures and Tables

**Table 1:** Explanation of designations used in Figure 1.

Designation	Explanation
**I**	Preform heating process and SBM process.
**II**	Hot filling process—carried out at a specially designed stand (Figure 2).
**1**	Delivery of the bottle on the waiting table (marked as 1 in Figure 2)—time from opening blow molds to starting the filling of hot water120 s; 90 manufactured bottles are numbered consecutively with a permanent marker, divided into three measuring series A, B, and C of 30 bottles each and put on the appropriate waiting table, with the first bottle for the A series, the second bottle for the B series, the third bottle for the C series, the fourth bottle for the A series, the fifth bottle for the B series, the sixth bottle for the C series, the seventh bottle for the A series, etc.
**2**	Rapid cooling of the bottle by spraying with cold water at 15 °C for 60 s (marked as 2 in Figure 2).
**3**	The maximum volume of a bottle measured by the gravimetric method (in accordance with PN-O-79782: 1996).
**4**	Taking pictures of the bottle.
**5**	Measurement of the position and shape of external and internal bottle markings applied to the preform-blow kinetics parameters (explained in Figure 6): a, p1, p2, p3, e, f.
**6**	Measurement of the bottle thickness profile by the FH4 inductive sensor (according to points arranged as in Figure 5).
**7**	Measurement of the bottle pressure resistance-using the CMC KUHNKE ABT-3100-PET pressure strength testing machine.
**8**	Cutting the sample out of bottles at the IV-2 thickness measurement point (base part of the bottle shown in Figure 5) for measuring the amount of oriented amorphous phase in accordance with formula (A36). The dimensions of the cut sample should be approximately 1 cm × 1 cm.
**9**	Filling the bottles with hot water at a temperature of 86 °C to the nominal volume level; hot water was delivered from a large tank with 86 °C water previously prepared in the tank (marked as 9 in Figure 2).
**10**	Free annealing (B series)—the bottles were filled to the nominal volume with hot water on the free heating place (marked as 10 in Figure 2) for 30 s.
**11**	Bath annealing (C series)—the filled bottles were placed in the 86 °C hot water bath (marked as 11 in Figure 2), the water level in the bath tank was the same as the water level in the bottle (elimination of pressure from the water filled inside the bottle reducing the shrinkage of the bottle because of microstructure changes).
**12**	Annealing time in the bath with hot water: 30 s.
**13**	Removing the bottles from the water bath.
**14**	Pouring out hot water from the bottles.
**A**	Bottles not annealed with hot water (A series).
**B**	Bottles annealed with hot water by free annealing (B series).
**C**	Bottles annealed with hot water by bath annealing (C series).
**D**	Technological parameters—general power of heating lamps in the preform heating oven.
**E**	Technological parameters—general power of electric heaters in blow mold.
**F**	Technological parameters—toughing time of the bottle surface in a hot blow mold.
**G**	Technological parameters—time from opening blow molds to start of filling process.
**H**	Technological parameters—temperature of hot water filled inside the bottle.
**I**	Technological parameters—annealing time of the bottle filled with hot water.

**Table 2:** The stretch blow moulding (SBM) process parameter values not subject to testing.

SBM Process Parameters			Heating Power of Individual Heating Lamps in the Heating Oven
Intrinsic viscosity of the raw material		0.7 dL/g	01: 55.0% 02: 18.0% 03: 12.0% 04: 13.0% 05: 21.5% 06: 0.0% 07: 0.0% 08: 0.0% 09: 0.0% General power of the oven: 65.0%
Stretching rod speed		1.2 m/s	
Initial blow start delays relative to the position of the stretching rod		55 mm	
Pre-blow air pressure		8.0 bars	
Pre-blow time		0.08 s	
Main blow air pressure		35 bar	
Main blow time		0.72 s	
Post-mold bottles cooling air temperature		19 °C	
Post-mold bottles cooling air pressure		2.5 bars	
Hot mold temperature profile (medium values)	thread area	21 °C	
	label area	125 °C	
	base area	62 °C	
Cold mold temperature		10 °C	

**Table 4:** Plan of a two-factor, bivalent experiment, where A factors have two levels (i = 1, 2), B factors have two levels (j = 1, 2), and “l” is the sample number for each level of A and B factors and varies from 1 to Nij.

NoE	Main Factors		Interaction Factors	Mean Response
	A	B	A∗B	$\tilde{y}$
1	-1	-1	1	$\bar{y_{1}}$
2	-1	1	-1	$\bar{y_{2}}$
3	1	-1	-1	$\bar{y_{3}}$
4	1	1	1	$\bar{y_{4}}$

**Table 5:** Plan for one one-factor, bivalent experiment for testing thickness profile, and blow kinetics coefficients (NoE—Number of Experiment).

NoE	Factor—SBM Process (Blow Mold Temperature)	Responses for Bottles in “A” Series					
		Thickness Profile			Blow Kinetics Coefficients		
		Point	Mean Value [mm]	Measurement Uncertainty [mm]	Coefficients	Mean Value	Measurement Uncertainty
**1**	-1 (cold mold)	I-1	0.23	0.03	I-w.a	1.46	0.03
		I-2	0.22	0.03	I-w.b	−0.59 mm	2.11 mm
		I-3	0.25	0.07	I-w.c	0.26 mm	1.23 mm
		II-1	0.16	0.01	I-w.d	3.16	0.79
		II-2	0.16	0.01	I-w.e	1.46	0.45
		II-3	0.16	0.01	I-w.f	1.69	0.42
		III-1	0.17	0.01	I-w.g	3.03	0.59
		III-2	0.17	0.01	II-w.a	2.02	0.03
		III-3	0.17	0.01	II-w.b	−0.56 mm	0.84 mm
		IV-1	0.19	0.01	II-w.c	0.61 mm	0.69 mm
		IV-2	0.19	0.01	II-w.d	3.17	0.17
		IV-3	0.19	0.01	II-w.e	1.81	0.33
		V-1	0.20	0.04	II-w.f	2.84	0.44
		V-2	0.21	0.04	II-w.g	3.19	0.20
		V-3	0.21	0.05	III-w.a	2.56	0.02
		-	-	-	III-w.b	0.14 mm	0.57 mm
		-	-	-	III-w.c	0.29 mm	0.60 mm
		-	-	-	III-w.d	3.12	0.19
		-	-	-	III-w.e	2.77	0.23
		-	-	-	III-w.f	3.88	0.24
		-	-	-	III-w.g	3.30	0.13
		-	-	-	IV-w.a	2.78	0.02
		-	-	-	IV-w.b	0.35 mm	0.48 mm
		-	-	-	IV-w.c	0.19 mm	0.33 mm
		-	-	-	IV-w.d	2.94	0.13
		-	-	-	IV-w.e	2.69	0.21
		-	-	-	IV-w.f	3.72	0.22
		-	-	-	IV-w.g	3.15	0.24
	1 (hot mold)	I-1	0.27	0.03	I-w.a	1.60	0.03
		I-2	0.25	0.03	I-w.b	0.19 mm	2.66 mm
		I-3	0.25	0.03	I-w.c	−0.55 mm	1.99 mm
		II-1	0.16	0.01	I-w.d	3.25	0.86
		II-2	0.16	0.01	I-w.e	1.48	0.36
		II-3	0.16	0.01	I-w.f	1.82	0.91
		III-1	0.18	0.01	I-w.g	3.11	1.01
		III-2	0.18	0.01	II-w.a	2.02	0.03
		III-3	0.18	0.01	II-w.b	0.16 mm	1.05 mm
		IV-1	0.20	0.01	II-w.c	0.07 mm	0.98 mm
		IV-2	0.19	0.01	II-w.d	4.00	0.53
		IV-3	0.19	0.01	II-w.e	2.04	0.27
		V-1	0.20	0.02	II-w.f	2.71	0.24
		V-2	0.19	0.02	II-w.g	4.09	0.34
		V-3	0.19	0.03	III-w.a	2.58	0.02
		-	-	-	III-w.b	−0.65 mm	0.93 mm
		-	-	-	III-w.c	−0.39 mm	1.26 mm
		-	-	-	III-w.d	3.65	0.34
		-	-	-	III-w.e	3.44	0.28
		-	-	-	III-w.f	4.33	0.32
		-	-	-	III-w.g	3.23	0.56
		-	-	-	IV-w.a	2.76	0.02
		-	-	-	IV-w.b	−0.29 mm	1.13 mm
		-	-	-	IV-w.c	0.44 mm	0.88 mm
		-	-	-	IV-w.d	3.11	0.39
		-	-	-	IV-w.e	3.06	0.31
		-	-	-	IV-w.f	4.04	0.48
		-	-	-	IV-w.g	3.17	0.42

**Table 6:** Plans for six one-factor, bivalent experiments for testing degree of crystallinity, density, and relaxation of the amorphous phase (NoE- Number of Experiment)—samples were cut out of bottles at IV-2 thickness measurement point (base part of the bottle shown in Figure 5), and cut out of preforms at four blow kinetics marks (shown in Figure 3).

NoE	Factor—SBM Process with Hot Fill Process			Results for Bottle Material					
	First Value (-1)	Second Value (1)		Density		DSC Crystallite		Relaxation of Amorphous Phase	
		SBM Process (Blow Mold Temperature)	Hot Fill Process	Mean [g/cm^3^]	Measurement Uncertainty [g/cm^3^]	Mean [%]	Measurement Uncertainty [%]	Mean [-]	Measurement Uncertainty [-]
2	preform	cold	A—lack	1.3578	0.0016	27.6	4.0	1.008	0.009
3	preform	cold	B—free	1.3589	0.0017	29.7	4.4	1.010	0.010
4	preform	cold	C—bath	1.3562	0.0017	30.8	4.1	1.014	0.010
5	preform	hot	A—lack	1.3664	0.0014	27.3	6.0	0.999	0.012
6	preform	hot	B—free	1.3660	0.0030	29.8	4.5	1.003	0.011
7	preform	hot	C—bath	1.3662	0.0033	29.1	4.6	1.002	0.011
**Results for preform material**				1.3385	0.0006	3.5	4.8	1.000	0.007

**Table 7:** Plans for nine one-factor, bivalent experiments for testing pressure resistance and thermal stability (NoE—Number of Experiment).

NoE	Factor—SBM Process (Blow Mold Temperature)		Factor—Hot Fill Process	
8	-1 (cold mold)	1 (hot mold)	A	
9	-1 (cold mold)	1 (hot mold)	B	
10	-1 (cold mold)	1 (hot mold)	C	
11	cold mold		-1 (A)	1 (B)
12	hot mold		-1 (A)	1 (B)
13	cold mold		-1 (A)	1 (C)
14	hot mold		-1 (A)	1 (C)
15	cold mold		-1 (B)	1 (C)
16	hot mold		-1 (B)	1 (C)

**Table 8:** Plans for three two-factor, bivalent experiments for testing pressure resistance and thermal stability (NoE—Number of Experiment).

Hot Fill Combination		Factors			Response			
Hot Fill	NoE	Mold Temperature	Hot Filling	Interaction Factor	Pressure Resistance		Shrinkage	
					Mean [bar]	Measurement Uncertainty [bar]	Mean [-]	Measurement Uncertainty [-]
A-B	17	-1 (cold)	-1 (A)	1	12.73	0.15	-	-
	18	-1 (cold)	1 (B)	-1	12.64	0.28	-	-
	19	1 (hot)	-1 (A)	-1	9.93	0.29	-	-
	20	1 (hot)	1 (B)	1	10.13	0.33	-	-
A-C	21	-1 (cold)	-1 (A)	1	12.73	0.15	-	-
	22	-1 (cold)	1 (C)	-1	12.75	0.24	-	-
	23	1 (hot)	-1 (A)	-1	9.93	0.29	-	-
	24	1 (hot)	1 (C)	1	10.21	0.18	-	-
B-C	25	-1 (cold)	-1 (B)	1	12.64	0.28	0.224	0.004
	26	-1 (cold)	1 (C)	-1	12.75	0.24	0.286	0.009
	27	1 (hot)	-1 (B)	-1	10.13	0.33	0.114	0.016
	28	1 (hot)	1 (C)	1	10.21	0.18	0.158	0.022

## Appendix A

Appendix A includes equations and figures that would disrupt the flow of the main text, but nonetheless remain crucial to understanding and reproducing the research shown.

(A1) $$\Delta_{t_{\mathrm{ij}}}=k\cdot \sqrt{{\left(\frac{\Delta_{1}}{\sqrt{3}}\right)}^{2}+\frac{\sigma_{\mathrm{ij}}{}^{2}}{n_{\mathrm{ij}}}}$$

where $\Delta_{t_{\mathrm{ij}}}$ is most likely the absolute error value for thickness measurements at the i-th measuring point (see Figure 5) of the j-th bottle for the assumed confidence level *p* = 0.95; k is the extension factor (for *p* = 0.95, k = 2 was assumed); $\sigma_{\mathrm{ij}}$ is the standard deviation from the average of measurements (if the distribution of results is a normal distribution, then the standard deviation from the mean as a measure of measurement error can be taken) at the i-th measuring point of the j-th bottle; $n_{\mathrm{ij}}$ is the number of measurement repetitions at the i-th measuring point of the j-th bottle; $\Delta_{1}$ is the measurement uncertainty of inductive sensor FH4 (Table 3). Equations (A2)–(A21) list the coefficients of the bottle blow kinetics for the j-th bottle.

(A2) $${w_{a}}_{\mathrm{ij}}=\frac{{a_{b}}_{\mathrm{ij}}}{{a_{p}}_{i}}\pm {\Delta_{w_{a}}}_{\mathrm{ij}}=\frac{a_{\mathrm{ij}}}{{a_{p}}_{i}}\pm {\Delta_{w_{a}}}_{\mathrm{ij}}.$$

(A3) $${\Delta_{w_{a}}}_{\mathrm{ij}}=\sqrt{{\left(\frac{\partial {w_{a}}_{\mathrm{ij}}}{\partial a_{\mathrm{ij}}}\cdot {\Delta_{a}}_{\mathrm{ij}}\right)}^{2}+{\left(\frac{\partial {w_{a}}_{\mathrm{ij}}}{\partial {a_{p}}_{i}}\cdot {\Delta_{a_{p}}}_{i}\right)}^{2}}=\sqrt{{\left(\frac{1}{{a_{p}}_{i}}\cdot {\Delta_{a}}_{\mathrm{ij}}\right)}^{2}+{\left(-\frac{a_{\mathrm{ij}}}{{{a_{p}}_{i}}^{2}}\cdot {\Delta_{a_{p}}}_{i}\right)}^{2}}$$

(A4) $${w_{b}}_{\mathrm{ij}}=\left(p1_{\mathrm{ij}}-p3_{\mathrm{ij}}\right)\pm \Delta_{{w_{b}}_{\mathrm{ij}}}$$

(A5) $$\Delta_{{w_{b}}_{\mathrm{ij}}}=\sqrt{{\left(\frac{\partial {w_{b}}_{\mathrm{ij}}}{\partial p1_{\mathrm{ij}}}\cdot {\Delta_{p1}}_{\mathrm{ij}}\right)}^{2}+{\left(\frac{\partial {w_{b}}_{\mathrm{ij}}}{\partial p3_{\mathrm{ij}}}\cdot {\Delta_{p3}}_{\mathrm{ij}}\right)}^{2}}=\sqrt{{\left({\Delta_{p1}}_{\mathrm{ij}}\right)}^{2}+{\left(-{\Delta_{p3}}_{\mathrm{ij}}\right)}^{2}}$$

(A6) $${w_{c}}_{\mathrm{ij}}=\left(a_{\mathrm{ij}}-p2_{\mathrm{ij}}\right)\pm {\Delta_{w_{c}}}_{\mathrm{ij}}$$

(A7) $${\Delta_{w_{c}}}_{\mathrm{ij}}=\sqrt{{\left(\frac{\partial {w_{c}}_{\mathrm{ij}}}{\partial a_{\mathrm{ij}}}\cdot {\Delta_{a}}_{\mathrm{ij}}\right)}^{2}+{\left(\frac{\partial {w_{c}}_{\mathrm{ij}}}{\partial p2_{\mathrm{ij}}}\cdot {\Delta_{p2}}_{\mathrm{ij}}\right)}^{2}}=\sqrt{{\left({\Delta_{a_{p}}}_{\mathrm{ij}}\right)}^{2}+{\left(-{\Delta_{p2}}_{\mathrm{ij}}\right)}^{2}}$$

(A8) $${w_{d}}_{\mathrm{ij}}=\frac{{d_{b}}_{\mathrm{ij}}}{{d_{p}}_{i}}\pm {\Delta_{w_{d}}}_{\mathrm{ij}}=\frac{p3_{\mathrm{ij}}-a_{\mathrm{ij}}}{{d_{p}}_{i}}\pm {\Delta_{w_{d}}}_{\mathrm{ij}}$$

(A9) $$\begin{array}{c} {\Delta_{w_{d}}}_{\mathrm{ij}}=\sqrt{{\left(\frac{\partial {w_{d}}_{\mathrm{ij}}}{\partial a_{\mathrm{ij}}}\cdot {\Delta_{a}}_{\mathrm{ij}}\right)}^{2}+{\left(\frac{\partial {w_{d}}_{\mathrm{ij}}}{\partial p3_{\mathrm{ij}}}\cdot {\Delta_{p3}}_{\mathrm{ij}}\right)}^{2}+{\left(\frac{\partial {w_{d}}_{\mathrm{ij}}}{\partial d_{p_{i}}}\cdot {\Delta_{d_{p}}}_{i}\right)}^{2}}=\\ =\sqrt{{\left(\frac{-1}{d_{p_{i}}}\cdot {\Delta_{a}}_{\mathrm{ij}}\right)}^{2}+{\left(\frac{1}{d_{p_{i}}}\cdot {\Delta_{p3}}_{\mathrm{ij}}\right)}^{2}+{\left(-\frac{{p3}_{\mathrm{ij}}-a_{\mathrm{ij}}}{{d_{p_{i}}}^{2}}\cdot {\Delta_{d_{p}}}_{i}\right)}^{2}} \end{array}$$

(A10) $${w_{e}}_{\mathrm{ij}}=\frac{{e_{b}}_{\mathrm{ij}}}{{e_{p}}_{i}}\pm {\Delta_{w_{e}}}_{\mathrm{ij}}=\frac{e_{\mathrm{ij}}}{{e_{p}}_{i}}\pm {\Delta_{w_{e}}}_{\mathrm{ij}}$$

(A11) $${\Delta_{w_{e}}}_{\mathrm{ij}}=\sqrt{{\left(\frac{\partial {w_{e}}_{\mathrm{ij}}}{\partial e_{\mathrm{ij}}}\cdot {\Delta_{e}}_{\mathrm{ij}}\right)}^{2}+{\left(\frac{\partial {w_{e}}_{\mathrm{ij}}}{\partial {e_{p}}_{i}}\cdot {\Delta_{e_{p}}}_{i}\right)}^{2}}=\sqrt{{\left(\frac{1}{{e_{p}}_{i}}\cdot {\Delta_{e}}_{\mathrm{ij}}\right)}^{2}+{\left(-\frac{e_{\mathrm{ij}}}{{{e_{p}}_{i}}^{2}}\cdot {\Delta_{e_{p}}}_{i}\right)}^{2}}$$

(A12) $${w_{f}}_{\mathrm{ij}}=\frac{{f_{b}}_{\mathrm{ij}}}{{f_{p}}_{i}}\pm {\Delta_{w_{f}}}_{\mathrm{ij}}=\frac{f_{\mathrm{ij}}}{{f_{p}}_{i}}\pm {\Delta_{w_{f}}}_{\mathrm{ij}}$$

(A13) $${\Delta_{w_{f}}}_{\mathrm{ij}}=\sqrt{{\left(\frac{\partial {w_{f}}_{\mathrm{ij}}}{\partial f_{\mathrm{ij}}}\cdot {\Delta_{f}}_{\mathrm{ij}}\right)}^{2}+{\left(\frac{\partial {w_{f}}_{\mathrm{ij}}}{\partial {f_{p}}_{i}}\cdot {\Delta_{f_{p}}}_{i}\right)}^{2}}=\sqrt{{\left(\frac{1}{{f_{p}}_{i}}\cdot {\Delta_{f}}_{\mathrm{ij}}\right)}^{2}+{\left(-\frac{f_{\mathrm{ij}}}{{{f_{p}}_{i}}^{2}}\cdot {\Delta_{f_{p}}}_{i}\right)}^{2}}$$

(A14) $${w_{g}}_{\mathrm{ij}}=\frac{{g_{b}}_{\mathrm{ij}}}{{g_{p}}_{i}}\pm {\Delta_{w_{g}}}_{\mathrm{ij}}=\frac{p1_{\mathrm{ij}}-p2_{\mathrm{ij}}}{{g_{p}}_{i}}\pm {\Delta_{w_{g}}}_{\mathrm{ij}}$$

(A15) $$\begin{array}{l} {\Delta_{w_{g}}}_{\mathrm{ij}}=\sqrt{{\left(\frac{\partial {w_{g}}_{\mathrm{ij}}}{\partial {p1}_{\mathrm{ij}}}\cdot {\Delta_{p1}}_{\mathrm{ij}}\right)}^{2}+{\left(\frac{\partial {w_{g}}_{\mathrm{ij}}}{\partial p2_{\mathrm{ij}}}\cdot {\Delta_{p2}}_{\mathrm{ij}}\right)}^{2}+{\left(\frac{\partial {w_{g}}_{\mathrm{ij}}}{\partial gp_{i}}\cdot {\Delta_{g_{p}}}_{i}\right)}^{2}}=\\ =\sqrt{{\left(\frac{1}{g_{p_{i}}}\cdot {\Delta_{p1}}_{\mathrm{ij}}\right)}^{2}+{\left(\frac{-1}{g_{p_{i}}}\cdot {\Delta_{p2}}_{\mathrm{ij}}\right)}^{2}+{\left(-\frac{{p1}_{\mathrm{ij}}-{p2}_{\mathrm{ij}}}{{g_{p_{i}}}^{2}}\cdot {\Delta_{g_{p}}}_{i}\right)}^{2}} \end{array}$$

(A16) $${\Delta_{a}}_{\mathrm{ij}}=k\sqrt{{\left(\frac{\Delta_{2}}{\sqrt{3}}\right)}^{2}+\frac{{{\sigma_{a}}_{\mathrm{ij}}}^{2}}{{n_{a}}_{\mathrm{ij}}}}$$

(A17) $${\Delta_{p1}}_{\mathrm{ij}}=k\sqrt{{\left(\frac{\Delta_{2}}{\sqrt{3}}\right)}^{2}+\frac{{{\sigma_{p1}}_{\mathrm{ij}}}^{2}}{{n_{p1}}_{\mathrm{ij}}}}$$

(A18) $${\Delta_{p3}}_{\mathrm{ij}}=k\sqrt{{\left(\frac{\Delta_{2}}{\sqrt{3}}\right)}^{2}+\frac{{{\sigma_{p3}}_{\mathrm{ij}}}^{2}}{{n_{p3}}_{\mathrm{ij}}}}$$

(A19) $${\Delta_{p2}}_{\mathrm{ij}}=k\sqrt{{\left(\frac{\Delta_{2}}{\sqrt{3}}\right)}^{2}+\frac{{{\sigma_{p2}}_{\mathrm{ij}}}^{2}}{{n_{p2}}_{\mathrm{ij}}}}$$

(A20) $${\Delta_{e}}_{\mathrm{ij}}=k\sqrt{{\left(\frac{\Delta_{7}}{\sqrt{3}}\right)}^{2}+\frac{{{\sigma_{e}}_{\mathrm{ij}}}^{2}}{{n_{e}}_{\mathrm{ij}}}}$$

(A21) $${\Delta_{f}}_{\mathrm{ij}}=k\sqrt{{\left(\frac{\Delta_{7}}{\sqrt{3}}\right)}^{2}+\frac{{{\sigma_{f}}_{\mathrm{ij}}}^{2}}{{n_{f}}_{\mathrm{ij}}}}$$

where j is the j-th bottle in the measurement series; $w_{a}$ is the relative axial displacement of the upper edge of the outer mark of the bottle relative to the position of the upper edge of the outer mark of the preform (the upper edge of the mark is the one closer to the thread); $w_{b}$ is axially shifting the bottom edge of the outer mark of the bottle relative to the bottom edge of the inner mark of the bottle; $w_{c}$ is axially shifting the top edge of the bottle’s internal mark to the top edge of the bottle’s outer mark; $w_{d}$ is the relative axial change in the length of the outer mark of the bottle relative to the axial length of the outer mark of the preform; $w_{e}$ is the relative circumferential change in the width of the outer mark of the bottle relative to the circumferential width of the outer mark of the preform; $w_{f}$ is the relative circumferential change of the inner mark width of the bottle relative to the circumferential inner mark width of the preform; $w_{g}$ is the relative axial change in the length of the inner mark of the bottle relative to the axial length of the inner mark of the preform; k is the extension factor (for p = 0.95, k = 2 was assumed); $n_{\mathrm{ij}}$ is the number of measurement repetitions at the i-th measuring mark of the j-th bottle; Δ is most likely the absolute error value for the assumed confidence level p = 0.95; σ is the standard deviation from the average of measurements; $\Delta_{2}$ is the electronic altimeter measurement uncertainty (Table 3); $\Delta_{7}$ is the electronic caliper measurement uncertainty (Table 3); $\Delta_{{a_{p}}_{i}}=\Delta_{{d_{p}}_{i}}=\Delta_{{e_{p}}_{i}}=\Delta_{{f_{p}}_{i}}=\Delta_{{g_{p}}_{i}}=0.02\;\mathrm{mm}$ is the numerically controlled lathe accuracy (Figure 3); i=1,2,3,4,5 is the i-th measuring mark (for the preform shown in Figure 3).

(A22) $$S_{B}=\frac{{V_{A}}_{\mathrm{msx}}-{V_{B}}_{\mathrm{msx}}}{{V_{A}}_{\mathrm{msx}}}\pm {\Delta_{S}}_{B}=\frac{\frac{M_{A}-m_{A}}{\rho_{T,p}}-\frac{M_{B}-m_{B}}{\rho_{T,p}}}{\frac{M_{A}-m_{A}}{\rho_{T,p}}}\pm {\Delta_{S}}_{B}=\frac{M_{A}-M_{B}-m_{A}+m_{B}}{M_{A}-m_{A}}\pm {\Delta_{S}}_{B}$$

(A23) $$\begin{array}{l} \Delta_{S_{B}}=\sqrt{{\left(\frac{\partial S_{B}}{\partial M_{A}}\cdot \Delta_{M_{A}}\right)}^{2}+{\left(\frac{\partial S_{B}}{\partial M_{B}}\cdot \Delta_{M_{B}}\right)}^{2}+{\left(\frac{\partial S_{B}}{\partial m_{A}}\cdot \Delta_{m_{A}}\right)}^{2}+{\left(\frac{\partial S_{B}}{\partial m_{B}}\cdot \Delta_{m_{B}}\right)}^{2}}=\\ =\sqrt{{\left(\frac{M_{B}-m_{B}}{{\left(M_{A}-m_{A}\right)}^{2}}\cdot {\Delta_{M}}_{A}\right)}^{2}+{\left(\frac{-1}{M_{A}-m_{A}}\cdot {\Delta_{M}}_{B}\right)}^{2}+{\left(\frac{m_{B}-M_{B}}{{\left(M_{A}-m_{A}\right)}^{2}}\cdot {\Delta_{m}}_{A}\right)}^{2}+{\left(\frac{1}{M_{A}-m_{A}}\cdot {\Delta_{m}}_{B}\right)}^{2}} \end{array}$$

(A24) $$S_{C}=\frac{{V_{A}}_{\mathrm{msx}}-{V_{C}}_{\mathrm{msx}}}{{V_{A}}_{\mathrm{msx}}}\pm {\Delta_{S}}_{C}=\frac{\frac{M_{A}-m_{A}}{\rho_{T,p}}-\frac{M_{C}-m_{C}}{\rho_{T,p}}}{\frac{M_{A}-m_{A}}{\rho_{T,p}}}\pm {\Delta_{S}}_{C}=\frac{M_{A}-M_{C}-m_{A}+m_{C}}{M_{A}-m_{A}}\pm {\Delta_{S}}_{C}$$

(A25) $$\begin{array}{l} \Delta_{S_{C}}=\sqrt{{\left(\frac{\partial S_{C}}{\partial M_{A}}\cdot \Delta_{M_{A}}\right)}^{2}+{\left(\frac{\partial S_{C}}{\partial M_{C}}\cdot \Delta_{M_{C}}\right)}^{2}+{\left(\frac{\partial S_{C}}{\partial m_{A}}\cdot \Delta_{m_{A}}\right)}^{2}+{\left(\frac{\partial S_{C}}{\partial m_{C}}\cdot \Delta_{m_{C}}\right)}^{2}}=\\ =\sqrt{{\left(\frac{M_{C}-m_{C}}{{\left(M_{A}-m_{A}\right)}^{2}}\cdot {\Delta_{M}}_{A}\right)}^{2}+{\left(\frac{-1}{M_{A}-m_{A}}\cdot {\Delta_{M}}_{C}\right)}^{2}+{\left(\frac{m_{C}-M_{C}}{{\left(M_{A}-m_{A}\right)}^{2}}\cdot {\Delta_{m}}_{A}\right)}^{2}+{\left(\frac{1}{M_{A}-m_{A}}\cdot {\Delta_{m}}_{C}\right)}^{2}} \end{array}$$

(A26) $$\Delta_{M_{A}}=k\cdot \sqrt{{\left(\frac{\Delta_{3}}{\sqrt{3}}\right)}^{2}+\frac{\sigma_{M_{A}}{}^{2}}{n_{M_{A}}}}$$

(A27) $$\Delta_{m_{A}}=k\cdot \sqrt{{\left(\frac{\Delta_{3}}{\sqrt{3}}\right)}^{2}+\frac{\sigma_{m_{A}}{}^{2}}{n_{m_{A}}}}$$

(A28) $$\Delta_{M_{B}}=k\cdot \sqrt{{\left(\frac{\Delta_{3}}{\sqrt{3}}\right)}^{2}+\frac{\sigma_{M_{B}}{}^{2}}{n_{M_{B}}}}$$

(A29) $$\Delta_{m_{B}}=k\cdot \sqrt{{\left(\frac{\Delta_{3}}{\sqrt{3}}\right)}^{2}+\frac{\sigma_{m_{B}}{}^{2}}{n_{m_{B}}}}$$

(A30) $$\Delta_{M_{C}}=k\cdot \sqrt{{\left(\frac{\Delta_{3}}{\sqrt{3}}\right)}^{2}+\frac{\sigma_{M_{C}}{}^{2}}{n_{M_{C}}}}$$

(A31) $$\Delta_{m_{C}}=k\cdot \sqrt{{\left(\frac{\Delta_{3}}{\sqrt{3}}\right)}^{2}+\frac{\sigma_{m_{C}}{}^{2}}{n_{m_{C}}}}$$

where $S_{B}$ is the relative shrinkage of a free annealed bottle; $S_{C}$ is the relative shrinkage of a bath annealed bottle; $M_{A}$ is the mass of the bottle fully filled with distilled water before the annealing process; $M_{B}$ is the mass of the bottle fully filled with distilled water after the free annealing process; $M_{C}$ is the mass of the bottle fully filled with distilled water after the bath annealing process; $m_{A}$ is the mass of the empty bottle before the annealing process; $m_{B}$ is the mass of the empty bottle after the free annealing process; $m_{C}$ is the mass of the empty bottle after the bath annealing process; n is the number of repetitions of measurements, the sample size; $\Delta_{3}$ is the electronic weighing uncertainty (Table 3); k is the extension factor (for p = 0.95, k = 2 was assumed); Δ is most likely the absolute error value for the assumed confidence level p = 0.95; σ is the standard deviation from the average of measurements; $\rho_{T,p}$ is the density of distilled water at the temperature and pressure of measurement [14].

(A32) $$C_{DSC}=\frac{\Delta h}{\Delta h_{f}{}^{o}}$$

(A33) $$C_{\rho }=\frac{\rho_{c}\cdot \left(\rho -\rho_{a}^{u}\right)}{\rho \cdot \left(\rho_{c}-\rho_{a}^{u}\right)}\cdot 100\%$$

(A34) CDSC≡Cρρau≡ρa⇒ρa=ρc·ρ·(1−CDSC)ρc−ρ·CDSC±Δρa

(A35) $$\begin{array}{c} \Delta_{\rho_{a}}=\sqrt{{\left(\frac{\partial \rho_{a}}{\partial \rho }\cdot \Delta_{\rho }\right)}^{2}+{\left(\frac{\partial \rho_{a}}{\partial C_{DSC}}\cdot \Delta_{C_{DSC}}\right)}^{2}}=\\ =\sqrt{{\left(\frac{\rho_{c}{}^{2}\cdot \left(1-C_{DSC}\right)}{{\left(\rho \cdot C_{DSC}-\rho_{c}\right)}^{2}}\cdot \Delta_{\rho }\right)}^{2}+{\left(\frac{\rho \cdot \rho_{c}\cdot \left(\rho -\rho_{c}\right)}{{\left(\rho \cdot C_{DSC}-\rho_{c}\right)}^{2}}\cdot \Delta_{C_{DSC}}\right)}^{2}} \end{array}$$

(A36) $$\Upsilon_{a}=\left(1-\frac{m_{a}-m_{a}^{u}}{m_{a}}\right)\cdot 100\%=\left(1-\frac{\rho_{a}\cdot V_{a}-\rho_{a}^{u}\cdot V_{a}}{\rho_{a}\cdot V_{a}}\right)\cdot 100\%=\frac{\rho_{a}^{u}}{\rho_{a}}\cdot 100\%\pm \Delta_{\Upsilon_{a}}$$

(A37) $$\Delta_{\Upsilon_{a}}=\sqrt{{\left(\frac{\partial \Upsilon_{a}}{\partial \rho_{a}}\cdot \Delta_{\rho_{a}}\right)}^{2}}=\sqrt{{\left(-\frac{\rho_{a}^{u}}{\rho_{a}{}^{2}}\cdot \Delta_{\rho_{a}}\right)}^{2}}\cdot 100\%$$

(A38) $$\Delta_{\rho }=k\cdot \sqrt{{\left(\frac{\Delta_{6}}{\sqrt{3}}\right)}^{2}+\frac{\sigma_{\rho }{}^{2}}{n_{\rho }}}$$

(A39) $$\Delta_{C_{DSC}}=k\cdot \sqrt{{\left(\frac{\Delta_{5}}{\sqrt{3}}\right)}^{2}+\frac{\sigma_{C_{DSC}}{}^{2}}{n_{C_{DSC}}}}$$

where $C_{\rho }$ is the weight crystallization degree of the polymer measured by density measurements, [%]; $C_{DSC}$ is the weight crystallization degree of the polymer measured by the DSC method, [%]; $\Upsilon_{a}$ is the weight relaxation degree of the amorphous phase, [%]; Δh is the specific melting heat of the crystalline phase in the PET sample, [J/(kg·K)]; $\Delta h_{f}{}^{o}$ is the heat of melting of the completely crystalline polymer in the same temperature range, [J/(kg·K)]; ρ is the measured PET sample density, [$g/{\mathrm{cm}}^{3}$]; $\rho_{a}^{u}$ is the density of the unoriented amorphous PET phase, $\rho_{a}^{u}=1.335\;g/{\mathrm{cm}}^{3}$ [37]; $\rho_{c}$ is the PET crystalline phase density (density of PET crystallites), $\rho_{c}=1.455\;g/{\mathrm{cm}}^{3}$ [37]; k is the extension factor (for *p* = 0.95, k = 2 was assumed); n is the number of repetitions of measurements, the sample size; Δ is most likely the absolute error value for the assumed confidence level *p* = 0.95; σ is the standard deviation from the average of measurements; $\Delta_{5}$ is the TA Inst Q20 microcalorimeter measurement uncertainty (Table 3); $\Delta_{6}$ is the uncertainty of density measurement in a gradient column filled with water solutions of calcium nitrate (Table 3).

(A40) $$\Delta_{b}=k\cdot \sqrt{{\left(\frac{\Delta_{4}}{\sqrt{3}}\right)}^{2}+\frac{\sigma_{b}{}^{2}}{n_{b}}}$$

where $\Delta_{b}$ is most likely the absolute error value of the pressure resistance measure of the entire bottle for the assumed confidence level *p* = 0.95; $\Delta_{4}$ is the uncertainty of measurement of pressure resistance testing machines (Table 3); k is the extension factor (for *p* = 0.95, k = 2 was assumed); n is the number of repetitions of measurements, the sample size; σ is the standard deviation from the average of measurements.

(A41) $${\left(\sigma_{e}{}^{2}\right)}_{t_{i}}=\max\left(\Delta_{t_{\mathrm{ij}}}{}^{2}\right)$$

(A42) $${\left(\sigma_{e}{}^{2}\right)}_{i}={\left(\sqrt{3}\cdot 0.95\cdot \frac{\Delta_{i}}{\sqrt{3}}\right)}^{2}={\left(0.95\cdot \Delta_{i}\right)}^{2}\;\mathrm{where}\;i=4,5,6$$

(A43) $${\left(\sigma_{e}{}^{2}\right)}_{w_{a_{i}}}=\max\left(\Delta_{w_{a_{\mathrm{ij}}}}{}^{2}\right)$$

(A44) $${\left(\sigma_{e}{}^{2}\right)}_{w_{b_{i}}}=\max\left(\Delta_{w_{b_{\mathrm{ij}}}}{}^{2}\right)$$

(A45) $${\left(\sigma_{e}{}^{2}\right)}_{w_{c_{i}}}=\max\left(\Delta_{w_{c_{\mathrm{ij}}}}{}^{2}\right)$$

(A46) $${\left(\sigma_{e}{}^{2}\right)}_{w_{d_{i}}}=\max\left(\Delta_{w_{d_{\mathrm{ij}}}}{}^{2}\right)$$

(A47) $${\left(\sigma_{e}{}^{2}\right)}_{w_{e_{i}}}=\max\left(\Delta_{w_{e_{\mathrm{ij}}}}{}^{2}\right)$$

(A48) $${\left(\sigma_{e}{}^{2}\right)}_{w_{f_{i}}}=\max\left(\Delta_{w_{f_{\mathrm{ij}}}}{}^{2}\right)$$

(A49) $${\left(\sigma_{e}{}^{2}\right)}_{w_{g_{i}}}=\max\left(\Delta_{w_{g_{\mathrm{ij}}}}{}^{2}\right)$$

(A50) $${\left(\sigma_{e}{}^{2}\right)}_{S}=\max\left\{\left[2{\left(\frac{M_{i}-m_{i}}{{\left(M_{A}-m_{A}\right)}^{2}}\right)}^{2}+2{\left(\frac{1}{M_{A}-m_{A}}\right)}^{2}\right]\cdot {\left(0.95\cdot \Delta_{3}\right)}^{2}\right\}\;\mathrm{where}\;i=A,B$$

(A51) $$\begin{array}{c} {\left(\sigma_{e}{}^{2}\right)}_{\gamma_{a}}=\max\left\{{\left(\frac{-\rho_{a}^{u}}{{{\rho_{a}}_{i}}^{2}}\right)}^{2}\left\{\begin{array}{c} {\left[\frac{\rho_{c}{}^{2}\left(1-{C_{\mathrm{DSC}}}_{i}\right)}{{\left(\rho_{i}{C_{\mathrm{DSC}}}_{i}-\rho_{c}\right)}^{2}}\right]}^{2}\cdot {\left(0.95\cdot \Delta_{6}\right)}^{2}+\\ +{\left[\frac{\rho_{i}\rho_{c}\left(\rho_{i}-\rho_{c}\right)}{{\left(\rho_{i}{C_{\mathrm{DSC}}}_{i}-\rho_{c}\right)}^{2}}\right]}^{2}\cdot {\left(0.95\cdot \Delta_{5}\right)}^{2} \end{array}\right\}\right\}\\ \mathrm{where}\;i=\mathrm{preform},\mathrm{bottle} \end{array}$$
